# Loneliness and global cognitive functioning in racially and ethnically diverse US midlife and older adults

**DOI:** 10.3389/fpsyg.2024.1344044

**Published:** 2024-06-19

**Authors:** David Camacho, Kelly Pacheco, Jerad Moxley, Maria P. Aranda, M. Carrington Reid, Elaine Wethington

**Affiliations:** ^1^Department of Disability and Human Development, University of Illinois Chicago, Chicago, IL, United States; ^2^Weill Cornell Medicine, Cornell University, New York, NY, United States; ^3^USC Suzanne Dworak-Peck School of Social Work, University of Southern California, Los Angeles, CA, United States; ^4^Edward R. Roybal Institute on Aging, University of Southern California, Los Angeles, CA, United States

**Keywords:** Alzheimer’s Disease and related dementias (ADRD), cognitive impairment (CI), perceived social isolation, minority aging, African American (AA), Hispanic and Latinx

## Abstract

**Introduction:**

Few studies have examined the association of loneliness and cognitive functioning in the US. We used two common measures of loneliness and examined their association in a large sample of US Black, Latino, and White adults (ages ≥ 50).

**Methods:**

We analyzed Wave 3 of the National Social Life, Health, and Aging Project (*N* = 2,757). We examined loneliness using one item from the CES-D and the Felt Loneliness Measure (NFLM); cognitive functioning was assessed using the Montreal Cognitive Assessment (MoCA) tool, where higher scores indicated better functioning. We used weighted ordinary least squares regressions to examine the effects of loneliness (CES-D loneliness and NFLM in separate models) on MoCA scores. In exploratory analyses, we examined if these relationships varied by race and ethnicity. We adjusted all models for sociodemographic and other salient factors (e.g., chronic disease, depressive symptoms, living alone).

**Results:**

Mean age was 63.49 years, 52% were female, and 9% were Black and 6% Latino persons. Approximately 54% endorsed feeling lonely on at least one measure; 31% (CES-D) and 46% (NFLM). The relationship between loneliness measures was positive and significant, *X*^2^ (1, *N* = 2,757) = 435.493 *p* < 0.001. However, only 40% of lonely individuals were identified as lonely on both assessments. CES-D loneliness was inversely (βˆ = −0.274, *p* = 0.032) associated with MoCA scores and this association did not vary by race and ethnicity. Greater NFLM loneliness was positively associated (βˆ = 0.445, *p* < 0.001) with higher MoCA scores for *Latino participants only.*

**Discussion:**

Loneliness appears to be an important predictor of cognitive functioning. However, the association of loneliness and cognitive functioning varied when using the CES-D loneliness item or the NFLM. Future work is needed to understand how loneliness and its clinically relevant dimensions (social, emotional, existential, chronicity) relate to global and individual cognitive domains. Research is needed with racially and ethnically diverse midlife and older adults, particularly to understand our counterintuitive finding for Latino participants. Finally, findings also support the need for research on interventions to prevent cognitive decline targeting loneliness.

## Introduction

Cognitive impairment disproportionately affects older adults, burdens systems of care, impairs quality of life, and constitutes an important research priority (World Health Organization, 2021). By 2060, the US population is projected to include 95 million older adults (Administration of Community Living, 2021). Of these, one third of individuals will be Black or Latino (Administration of Community Living, 2020a,b). Further, Blacks and Latino individuals are more likely to experience cognitive impairment compared to their White counterparts (Alzheimer’s Association, 2016). Therefore, there is a critical need to elucidate determinants and modifiable factors (e.g., loneliness) that impact cognitive health (Peterson et al., 2020; Aranda et al., 2023). However, these examinations should consider common contextual challenges experienced by Black and Latino persons in the US.

Loneliness refers to the distressing feeling that occurs when individuals perceive their social needs are not met by the quantity and particularly the quality of their social relationships (Perlman and Peplau, 1981; Hawkley and Cacioppo, 2010). Loneliness is different from but may coincide with social isolation, a depressive episode, or both. Social isolation is the absence or limited number of social relationships (de Jong-Gierveld et al., 2006; Perissinotto and Covinsky, 2014), while the primary symptoms of clinical depression are low mood (e.g., sadness) and lack of interest or pleasure in activities (Uher et al., 2014).

Loneliness may occur at any age. However, limitations in later life can limit opportunities to engage in socialization activities, but they may also shield against loneliness (Carstensen, 1992; Baltes and Carstensen, 2003). For example, older adulthood increases the likelihood of health challenges (e.g., chronic diseases; Vetrano et al., 2018), and frailty (Bandeen-Roche et al., 2015) that may diminish physical functioning and restrict social integration (Hoogendijk et al., 2016). Older adulthood also increases the likelihood of loss of significant others, reducing the pool of people they may count on. However, older adults’ expectations of chronic disease, lowered physical functioning, and social loss may promote compensatory mechanisms, including focusing on fewer but higher quality relationships (Carstensen, 1992; Baltes and Carstensen, 2003).

Loneliness is an important predictor of poor health outcomes (e.g., Ong et al., 2016), including poorer cognitive functioning around the world (Boss et al., 2015; Lara et al., 2019). However, the pathways between loneliness and cognitive functioning are not well understood (Boss et al., 2015). Loneliness may impact cognitive functioning via prolonged activation of the hypothalamus–pituitary–adrenal (HPA) axis, which has been associated with hypercortisolism as well as chronic psychological stress and loneliness (Dallman et al., 2004; Steptoe et al., 2004; Adam et al., 2006). Compared to those without loneliness, individuals experiencing loneliness report more chronic stressors (Hawkley and Cacioppo, 2007) and are more likely to perceive daily events as stressful (Cacioppo, 1994). Prolonged hypercortisolism may lead to cortical cellular damage and altered cognitive function in the form of dementia (Epel, 2009).

Despite growing interest, there are multiple gaps in understanding the relationship between loneliness and cognitive functioning. First, experiences and management of loneliness and cognitive health may be shaped by context, including variations in healthcare resources and cultural perspectives (e.g., Rokach, 2018; Kerwin et al., 2022). To date, only a limited number of studies have focused on US samples (Boss et al., 2015; Lara et al., 2019).

Five US-based studies have examined data from the Health and Retirement Study (HRS). Two HRS studies explored loneliness using a one-item measure of loneliness from the CES-D. Donovan et al. (2017) examined individuals 65 years and older and found that loneliness accelerated cognitive decline over a 12-year period even after accounting for relevant covariates including depressive symptoms. Similarly, Yu et al. (2023) examined participants 50 years and older using the 1996–2016 HRS data and found that “cumulative loneliness” (increasing number of waves a participant acknowledged loneliness) was negatively associated with memory function.

Two additional HRS studies used the 3-item UCLA loneliness scale. Griffin et al. (2020) examined HRS participants 65 years and older. Their cross-sectional analyses indicated that loneliness was inversely associated with total global cognitive functioning based on the Telephone Interview for Cognitive Status (TICS). However, their 6-year longitudinal analyses indicated no significant relationship between loneliness and cognitive decline. Sutin et al. (2020) examined participants 50 years and older over a period of 10 years, where dementia (TICS score of less than six) was the outcome of interest. Their results indicated that for every one-point increase in loneliness, the risk of developing dementia increased by 40%. The authors noted similar results when using the CES-D loneliness measure (“Much of the time during the past week you felt lonely”). Further, their results did not vary by race and ethnicity (i.e., African American, Hispanic, White individuals).

Poey et al. (2017) examined data from the Aging, Demographics, and Memory Study, which randomly enrolled individuals who participated in the 2000 and 2002 HRS waves. They also used the one-item measure of loneliness from the CES-D. Findings indicated that loneliness moderated the relationship between APOE e4 allele status and cognitive impairment based on nurse and neuropsychology technician assessments. Compared to individuals without APOE e4 allele or loneliness, those with the APOE e4 allele only were three times more likely to experience cognitive impairment. However, those with both the APOE e4 allele and loneliness were five times more likely to experience cognitive impairment.

Two studies by Wilson et al. (2007, 2015) used the De Jong-Giervald Loneliness Scale and analyzed data from the Rush Memory and Aging Project in a sample with a mean baseline age of 80.3 years. Wilson et al. (2007) found that at baseline, loneliness was associated with worse cognitive functioning (based on their composite measure of 19 different cognitive tests). After 4 years, loneliness was positively associated with more rapid cognitive decline. Wilson et al. (2015) found that loneliness did not moderate the relationship between negative social interactions and mild cognitive impairment (MCI).

Pluim et al. (2023) studied the relationship of loneliness, purpose in life, and subjective cognitive decline (SCD) from a subsample of data collected online during the COVID-19 pandemic with US and Latin American adults. Their subsample focused on US Asian, Black, Latino, and White adults with a mean age of 67 years and high levels of education (mean: 17.1 years of education, SD = 3.2). Loneliness was measured by the De Jong-Giervald Loneliness Scale. In adjusted models (e.g., controlling for sociodemographic factors, living arrangement, occupation status, etc.), they found that loneliness increased the likelihood of reporting SCD in White participants only.

Two studies examined the relationship of loneliness and cognitive functioning using the National Social Life, Health, and Aging Project (NSHAP). Kim et al. (2020) followed participants (mean age: 69 years) from Wave 1 across 10-year period to examine mediating factors in the relationship between loneliness and cognitive functioning. They used the 3-item version of the UCLA Loneliness scale and the Montreal Cognitive Assessment (MoCA). The study findings suggest that loneliness may indirectly affect cognitive functioning as their adjusted models revealed that functional ability, self-rated health, and depressive symptoms significantly mediated the effects?

Ishikawa et al. (2022) examined the cross-sectional relationship between perceived isolation and MCI using Wave 3 of the NSHAP. The investigators focused their examination on Black and White participants (95% of sample) and did not distinguish if individuals identified as Hispanic or Latino. Perceived isolation included questions about emotional and instrumental support from family members, friends, and spouse or partner as well as the 3-item UCLA loneliness scale. Ishikawa et al. used the MoCA cutoff of 23 to indicate possible MCI. They found that after controlling for demographic factors only, individuals with perceived isolation were more likely to experience MCI. Of note, neither Kim et al. (2020) nor Ishikawa et al. (2022), examined whether the relationship between loneliness and cognitive functioning varied across racial and ethnic groups.

A second critical issue in the study of loneliness and cognitive functioning is the variability in the assessment of loneliness (Boss et al., 2015; Lara et al., 2019). There are multiple measures of loneliness (Maes et al., 2022) and no consensus or gold standard scale. Extant studies (Boss et al., 2015; Lara et al., 2019) tend to use either “direct” assessments that include the term “lonely” (e.g., CES-D item “I feel lonely”) or “indirect” measures that avoid the use of the term (e.g., the 3-item UCLA scale; Shiovitz-Ezra and Ayalon, 2012). Some studies have found low concordance between the CES-D (direct assessment) and the dichotomized three-item UCLA (indirect assessment) and note that these assessments may capture different groups of individuals living with loneliness (e.g., Victor et al., 2005; Shiovitz-Ezra and Ayalon, 2012). More recently, Newmyer et al. (2021) analyzed data from 10 countries and concluded that both measures are valid measures of loneliness. They also found that the two measures function similarly across gender and age groups. However, they raised the possibility that there could be cross-cultural and contextual variation in the validity of loneliness measures (which they were unable to explore). Finally, current studies provide limited insight into possible differences in measures and their relationship with cognitive functioning measures.

Critical theoretical factors may contribute to measures capturing different groups living with loneliness. For example, stigma (Crocker and Major, 1989; Lau and Gruen, 1992) associated with the experience of loneliness may lead some individuals to not report their true feelings when using the direct assessment approach. Direct assessment (e.g., CES-D item) explores loneliness over the past week, while the 3-item UCLA (and its derivatives) examines a general feeling over an undefined timeframe (Shiovitz-Ezra and Ayalon, 2012). Further, we underscore that the impact of stigma and disclosure of loneliness may different and even be magnified in minoritized groups (Black and Latino groups). Despite these important theoretical differences, only one US study (Sutin et al., 2020) has explored the relationship between loneliness and cognitive functioning using both direct and indirect assessments. Sutin and colleagues found similar results using the 3-item UCLA scale and the one-item CES-D loneliness assessment. However, it is unclear if these results will be the same across other large datasets.

A third critical issue is that few US studies have examined loneliness in midlife and older Black or Latino individuals (Ojembe et al., 2022; Tibirica et al., 2022), or if the relationship between loneliness and cognitive functioning varies by race and ethnicity (e.g., Pluim et al., 2023). These studies have linked loneliness to health outcomes (e.g., frailty, cardiovascular disorders, self-rated health), including lower cognitive functioning (Gerst-Emerson et al., 2014; Han et al., 2017; Estrella et al., 2021). However, these studies focused on older Black adults living with HIV (Han et al., 2017), another focused on Mexican Americans over the age of 80 years, and none examined if the relationship between loneliness and cognitive functioning varied as a function of race and ethnicity.

Review articles that have considered the health impact of stressor exposure on physical and mental health (Ferraro and Shippee, 2009; Forrester et al., 2019) provide a strong basis for considering the cumulative and joint effect of stressful experiences such as loneliness on cognitive health. For example, low levels of education and low income are positively associated with the presence of loneliness and poorer cognitive functioning (e.g., Theeke, 2010; Chen et al., 2014; Weuve et al., 2018; Díaz-Venegas et al., 2019). Because US Black and Latino individuals are more likely to experience exposure to socioeconomic challenges that are associated with higher levels of stress exposure (e.g., low education, poverty) compared to their White counterparts (Ferraro and Shippee, 2009; Forrester et al., 2019) and because loneliness enhances stressful perceptions of daily events or challenges (Cacioppo et al., 2006; Hawkley and Cacioppo, 2010), we consider that the reciprocal relationship between exposure to risks and limited opportunities and resources, and loneliness may contribute to prolonged and higher levels of stress among Black and Latino persons across the life course. The higher frequency of stress and its biological impact could potentially accelerate cellular damage in the brain and lead to poorer cognitive functioning among midlife and older Black and Latino individuals compared to White participants (Epel, 2009; Zaheed et al., 2020). Therefore, we posit that the impact of loneliness on cognition may vary as a function of race and ethnicity, with Black and Latino individuals being more vulnerable to the effects of loneliness compared to their White counterparts.

In sum, these results highlight a need for further work that examines the relationship between loneliness and cognitive functioning in the US. Most but not all studies have noted an inverse relationship between loneliness and cognitive functioning. In this study, our primary aim was to examine the effects of loneliness on cognitive functioning in US Black, Latino, and White midlife and older adults (ages ≥ 50) from the NSHAP. Our sample included individuals who completed both the CES-D loneliness item and the 3-item NSHAP Felt Loneliness Measure (NFLM), as well as the MoCA at Wave 3 conducted in 2015 (Payne et al., 2014; Shega et al., 2014). As a secondary aim, we examined if the relationship between loneliness and cognition varies as a function of assessment type. Finally, we note that limited attention has been paid to how the relationship of loneliness and cognition varies across racial and ethnic groups. We conduct exploratory analyses that examine how the relationship of loneliness and cognition may vary by race and ethnicity. Guided by prior literature and theory, we posit that loneliness will contribute to poorer cognitive functioning. More specifically, we hypothesize that both assessments of loneliness will be inversely associated with cognitive functioning. Finally, we hypothesized that Black and Latino individuals with loneliness will experience poorer cognitive functioning compared to their White counterparts with the same levels of loneliness.

## Materials and methods

### Data source

The NSHAP is a nationally representative survey of midlife and older adults living in the community and is designed to assess the physical, mental, and social well-being of home-dwelling midlife and older Americans (O’Muircheartaigh et al., 2021). We analyzed data from Wave 3 (*n* = 4,777, collected 2015–2016), which included in-person interviews from two cohorts: (1) respondents continuing from the first rounds of interviews (born 1920–1947) and (2) newly recruited participants (born 1948–1965). Live-in partners of both cohorts were also eligible for the interviews. In addition to the in-person interviews, participants were asked to complete leave-behind, self-administered questionnaires and up to 11 biomeasures. We analyzed data from only Wave 3 for two reasons. First, the measures of cognitive functioning and loneliness in the planned analyses are present in Wave 3. The leave-behind questionnaire also included concepts of theoretical interest (e.g., community participation, perceived discrimination). Second, the inclusion of a fresh sample of midlife and older adults in Wave 3, as well as live-in partners, increased the sample size available to test study hypotheses and increased the age range of the sample to include the baby boomer cohort. Final return rates for the leave-behind questionnaire were satisfactory: 85% for the full sample, 91% for the continuing participants, and 80% for the newly recruited participants (O’Doherty et al., 2021). Data were collected in English and Spanish by the University of Chicago’s National Opinion Research Center.

### Population

Our target population included home-dwelling adults aged 50 and older who completed all items of our cognitive measure and were Black, Latino, or White persons. Our final weighted sample included 2,757 individuals who were either White (*n* = 2,371), Black (*n* = 240) or Latino (*n* = 146).

### Measurements

#### Outcome

##### Global cognitive functioning

The Chicago Cognitive Functions Measure (CCFM) was used to assess multiple cognitive domains, including (1) orientation, (2) naming, (3) executive functioning, (4) visuo-construction, (5) memory, (6) attention, (7) language, and (8) abstraction (Shega et al., 2014). We examined global CCFM scores with a possible range from 0 to 20, where higher scores indicate overall better cognitive function. Following Shega et al.’s (2014) approach, we converted CCFM scores into MoCA scores using the formula MoCA = (1.14 × CCFM) + 6.83. The MoCA has been used with a variety of populations and racially diverse samples (e.g., Rossetti et al., 2011; Zhou et al., 2015). We examined MoCA as a continuous score. Although prior studies have used a standard cutoff of 26 (Nasreddine et al., 2005) to indicate possible cognitive impairment, we considered that cutoff may vary as a function of race and ethnicity and education (Rossetti et al., 2011; Zhou et al., 2015). Because there is no accepted cutoff score for Black and Latino persons, we limited using cutoff score of 26 to descriptive purposes. For our primary analyses, we examined the MoCA as a continuous score.

##### Main predictors

We examined loneliness using two separate measures. First, we took one item (“I feel lonely”) from the CES-D that assessed the frequency of depressive symptoms in the past week (0 = rarely or none of the time, 1 = some of the time, and 2 = much or most of the time; Hawkley et al., 2006). Similar to prior research (Cornwell and Waite, 2009), we dichotomized responses by recoding all participants who responded some, much or most of the time as “lonely” (1 = Yes).

We also assessed loneliness using the NFLM. The NFLM is very similar to the 3-item UCLA loneliness scale. The NFLM assessed perceived frequency of lack of companionship, feeling left out, and feeling isolated, with possible responses of 0 = never, 1 = hardly ever, 2 = some of the time, and 3 = often. We followed scoring recommendations from Payne et al. (2014) on use of the NSHAP data. In line with their recommendations, we combined “never” and “hardly ever.” Total scores ranged from 0 to 6, with higher scores reflecting greater levels of loneliness. We also used their recommended cutoff of 1 to determine the presence of loneliness.

##### Covariates

In our analyses, we considered common social and health factors that contribute to poorer cognitive functioning. In our models we included available NSHAP measures indicative of individual *cumulative inequality* factors (education, perceived economic status, skipping healthcare due to in adequate health insurance, perceived discrimination) to capture their current direct effects on cognitive functioning (Ferraro and Shippee, 2009; Forrester et al., 2019). However, available measures do not fully capture the complexity of these experiences, including the magnitude, onset, or duration of exposure to these factors and their associated advantages and disadvantages across the life course and social systems. Therefore, we use the NSHAP-recommended categories of race and ethnicity to capture overall group differences resulting from cumulative inequality.

*Race and ethnicity* was assessed with two questions: “Do you consider yourself primarily White or Caucasian, Black or African American, American Indian, Asian, or something else?” and “Do you consider yourself Hispanic or Latino?” To analyze by group, we use an NSHAP-coded race and ethnicity variable that classified participants into four mutually exclusive groups: (1) non-Hispanic White, (2) Black (which included Hispanics who self-reported Black race), (3) Hispanic (of all races except “Black”), and (4) “other” (e.g., Asian, Native American, Pacific Islander).

Lower levels of education and low income have been associated with the presence of loneliness and poor cognitive functioning in US samples of midlife and older adults (Sachs-Ericsson et al., 2009; Theeke, 2010; Zhang et al., 2020). Further, access to healthcare (e.g., having adequate insurance) may contribute to better preventive care that in turn may improve health outcomes (McMaughan et al., 2020).

*Educational attainment* was measured as 1 = less than high school; 2 = high school or equivalent; 3 = vocational certificate, some college or associate degree, and 4 = bachelor’s degree or more.

*Perceived economic position* was determined by asking participants: “Compared with American families in general, would you say that your household income is 1 = far below average; 2 = below average; 3 = average; 4 = above average; or 5 = far above average?” Answers ranged from 1 to 5. We examined this variable as a continuous score, with higher scores indicating higher perceived economic position.

*Regarding Inadequate health insurance:* participants reported whether they had difficulty in receiving healthcare services because of a lack of adequate insurance: (1) “In the past year, has a lack of adequate health insurance kept you from getting medical care?” and (2) “In the past year, has a lack of adequate health insurance kept you from getting prescription medications?” Possible responses were yes or no. We recoded these items into one variable. If participants answered affirmatively to either original item, then they were coded as yes.

We also note that discrimination has detrimental effects on health including cognitive functioning (Pascoe and Smart Richman, 2009; Barnes et al., 2012). *Perceived discrimination* was measured by an adapted version of the Perceived Discrimination Scale (Williams et al., 1997; Monk et al., 2021). The two items included: “In your day-to-day life, how often have you been treated with less courtesy than other people?” and “In your day-to-day life, how often have people acted as if they are better than you are?” Response options were 0 = never, 1 = less than once a year, 2 = about once or twice a year, 3 = several times a year, 4 = about once a month, 5 = every week, and 6 = several times a week. We summed both items to create a total score (range: 0–12).

Age, biological sex, and marital status are important predictors of cognitive decline and impairment (Ferreira et al., 2014; Murman, 2015; Liu et al., 2019).

*Sociodemographic variables* included age (in years), sex (0 = male, 1 = female), and marital status (married or living as 1 = married, divorced, separated, or never 2 = married and 3 = widowed).

We considered that both chronic conditions (Maresova et al., 2019) and depressive symptoms (Rock et al., 2014) may contribute to poor cognitive functioning. The *total number of chronic diseases* was calculated by responses to separate items with a stem asking, “Has a medical doctor ever told you that you have: heart disease, arthritis, breathing problems, stroke, hypertension, diabetes, and cancer?” The total score ranged from 0 to 7.

*Depressive symptoms* were measured by the NSHAP Depressive Symptoms Measure (NDSM). The 11-item NDSM was derived from the well-validated CES-D depression instrument (Radloff, 1977; Payne et al., 2014). Similar to the original CES-D, the NDSM assesses the frequency of depressive symptoms in the past week. Original responses included 0 = rarely or none of the time, 1 = some of the time, 2 = occasionally, and 3 = most of the time. We followed the suggestion and recoded original responses of Payne et al. (2014) into three categories (0 = rarely or none of the time, 1 = some of the time, and 2 = much or most of the time). We did not use the item “I feel lonely” as this was already used in our study as a measure of loneliness (Hawkley et al., 2006). We summed scores for the remaining 10 items (possible range: 0–20).

We also considered that objective measures of social relations may also contribute to cognitive impairment (Evans et al., 2019). We did not have a direct measure of social isolation. However, we included in our models two related concepts that capture quantity of social relationships (Wang et al., 2017). *Community participation* during the past 12 months was assessed via three items examining frequency of volunteer work, attendance of social meetings, and gatherings with friends or relatives (Cornwell and Waite, 2009). Possible responses were 0 = never, 1 = less than once a year, 2 = about once or twice a year, 3 = several times a year, 4 = about once a month, 5 = every week, and 6 = several times per week. We created a sum score for these three items (possible range: 0–18).

A household roster was not available in the analysis dataset. Therefore, *living alone* was based on an examination of individuals’ reported social networks. Questions asked whether each person listed in individuals’ networks lived in their home. We identified individuals as living alone if they reported that nobody in their social network was living in their residence.

### Analytical strategy

Our primary goal was to examine the relation between loneliness and global cognitive functioning. We first examined descriptive statistics for all participants. We also examined Pearson’s correlations between primary predictors and MoCA scores (Table 1).

**Table 1:** Weighted descriptive and bivariate statistics at wave 3 (*n* = 2,757).

	Total	Bivariate relationship with MoCA score
	Mean and SD, or proportion	βˆ, SE, *p*-value
Global cognitive score	24.80(3.18)	N/A
**Loneliness**		
Lonely on CES-D	0.30	−0.934 (0.126), *p* < 0.001
Lonely NFLM score	1.11 (1.51)	−0.161(0.038), *p* < 0.001
**Race and ethnicity**		
Black	0.09	−2.755 (0.200), *p* < 0.001
Latino	0.06	−2.231(0.252), *p* < 0.001
White	0.85	Reference
Age	63.49 (9.2)	−0.061(0.007), *p* < 0.001
**Sex**		
Female	0.52	0.369(0.118), *p* < 0.001
**Marital status**		
Married or living with partner	0.72	Reference
Divorced, separated, or never married	0.19	−0.477(0.146), *p* < 0.001
Widowed	0.09	−1.430(0.213), *p* < 0.001
**Educational attainment**		
Bachelors or more	0.33	Reference
Vocational certificate, some college, or associate degree	0.38	−1.403(0.129), *p* < 0.001
High school or equivalent	0.22	−2.302(0.150), *p* < 0.001
Less than high school	0.07	−4.173 (0.238), *p* < 0.001
**Perceived economic position**	3.03 (0.97)	0.853(0.058), *p* < 0.001
**Inadequate Health Insurance**		
Yes	0.14	−0.478(0.167), *p* < 0.004
**Chronic disease total**	1.33 (1.21)	−0.310 (0.048), *p* < 0.001
Depressive symptoms	7.59 (3.08)	−0.163 (0.019), *p* < 0.001
Community participation	9.21 (4.22)	0.124(0.014), *p* < 0.001
Perceived discrimination	2.87 (2.73)	0.022(0.021), *p* = 0.304
Living alone	0.23	392 (0.135), *p* < 0.004

In our primary models (Table 2), the dependent variable was MoCA score. To minimize the potential for confounding (Hawkley et al., 2006), we examined a priori main effects and entered our loneliness measures into separate models. Model 1 included CES-D loneliness (yes, no) and Model 2 included the NFLM loneliness score. All adjusted models included race and ethnicity (Black, Latino, White), sex (male, female), marital status (married or living with partner: divorced, separated, or never married; and widowed), educational attainment (bachelor’s or more; vocation certificate, some college, or associate degree; high school or equivalent; and less than high school), skipping health services (no, yes), and living alone as fixed classification factors. Covariates included NFLM loneliness score, age (in years), household income (1–5), total number of chronic medical conditions (0–7), perceived discrimination score (0–14), depressive symptoms (0–20), and community participation (0–18).

**Table 2:** Effects of loneliness, and race/ethnicity on global cognitive functioning.

	Model 1	Model 2
	Lonely on CES-D	Lonely NFLM score
	βˆ, SE, *p*-value	βˆ, SE, *p*-value
**Loneliness**		
Lonely on CES-D	−0.275(0.128), *p* < 0.032	N/A
Lonely NFLM score	N/A	−0.001(0.038), *p* = 0.989
**Race and ethnicity**		
Black	−0.2.703(0.184), *p* < 0.001	−0.2.704(0.184), *p* < 0.001
Latino	−1.816(0.231), *p* < 0.001	−1.833(0.231), *p* < 0.001
White	Reference	Reference
Age	−0.063(0.007), *p* < 0.001	−0.063(0.007), *p* < 0.001
**Sex**		
Female	0.528(0.106), *p* < 0.001	0.519(0.106), *p* < 0.001
**Marital status**		
Married or living with partner	References	References
Divorced/separated/never	0.127(0.135), *p* = 0.347	0.073(0.136), *p* = 0.592
Widowed	−0.399(0.204), *p* = 0.051	−0.472(0.202), *p* < 0.020
**Educational attainment**		
Bachelors or more	References	References
Voc cert/some college/assoc.	−0.959(0.126), *p* < 0.001	−0.947(0.126), *p* < 0.001
High school or equivalent	−1.657 (0.151), *p* < 0.001	−1.653 (0.151), *p* < 0.001
Less than high school	−3.106(0.238), *p* < 0.001	−3.091(0.238), *p* < 0.001
**Perceived economic position**	0.320(0.062), *p* < 0.001	0.325(0.062), *p* < 0.001
**Inadequate Health Insurance**		
Yes	0.152(0.154), *p* = 0.324	0.159(0.154), *p* = 0.302
**Chronic disease total**	0.019(0.044), *p* = 0.666	0.016(0.044), *p* = 0.724
Depressive symptoms	−0.086(0.020), *p* < 0.001	−0.103(0.019), *p* < 0.001
Community participation	0.033(0.013), *p* < 0.013	0.035(0.013), *p* < 0.010
Perceived discrimination	0.023(0.021), *p* = 0.266	0.025(0.021), *p* = 0.234
Living alone	0.132(0.120), *p* = 0.274	0.139(0.120), *p* = 0.247

To support interpretation, we examined differences between loneliness groups. We used a cutoff score of 1 (Payne et al., 2014) to identify individuals who were lonely based on the NFLM. First, we conducted chi-square test between our dichotomized loneliness measures. Second, we created four groups (no loneliness on either measure, CES-D lonely only, NFLM lonely only, lonely on both measures). We used logistic regression to examine the differences across the groups of CES-D lonely only and NFLM lonely only. These groups represented individuals who were inconsistently classified as lonely across assessments.

**Figure 1:**
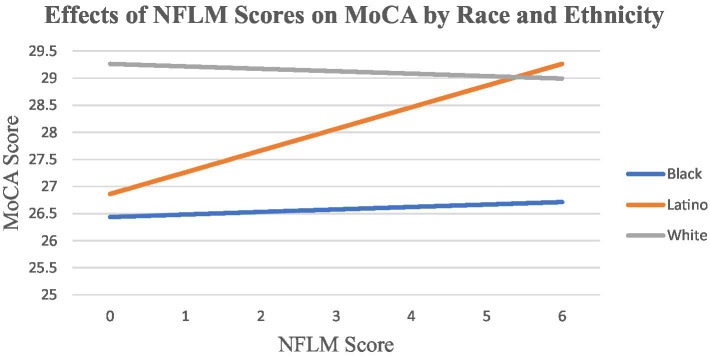
Effects of NFLM scores on MoCA by race and ethnicity.

We considered that disproportionate exposure to risks, opportunities, and resources (Ferraro and Shippee, 2009) across the life course may contribute to varying impact of loneliness on stress and cognitive functioning. We conceptualized racial and ethnic groups as categories that encapsulate the overall differences in exposure to risk or opportunity. In our models, we controled for multiple factors that may contribute to cumulative inequality in health outcomes, including educational attainment, perceived economic position, skipping health services, and perceived discrimination. However, these available NSHAP measures do not fully capture all factors and experiences that may contribute to disproportionate exposure to risk or opportunities (e.g., quality of education, wealth, quality of healthcare, etc.). To test differences in the impact of loneliness on cognitive functioning across race and ethnicity, we systematically examined the interactions of each loneliness measure (CES-D and NFLM) and race and ethnicity. In these models, we controlled for exposure to risk or opportunity, sociodemographics, and health factors. We demonstrate significant findings in Figure 1.

We used ordinary least squares regressions. We applied NSHAP-generated person-level weights that accounted for non response to all statistical analyses (detailed descriptions of weighting approach is found in O’Muircheartaigh et al., 2021). We used the recommended Wave 3 NSHAP variable (weight_adj) that assigns each case different weights (by simulated replication) and is designed to provide unbiased estimates of population parameters IBM SPSS Statistics (Version 29) (O’Muircheartaigh et al., 2021). Assigned weights indicated the number of observations that each case represented. Cases with missing values were excluded from our analyses. We also conducted analyses using linear mixed models to control for nesting among subjects from the same household. We performed analyses with SPSS version 27 survey procedures.

## Results

We present results for our original non nested models. Our nested analyses supported the robustness of our primary and exploratory findings. Most of our results were the same, with one exception. We describe this difference in its appropriate section. Nested models are available upon request.

### Descriptive statistics

Table 1 demonstrates the descriptive information of our weighted sample of 2,757 NSHAP participants. The mean MoCA score was 24.80 (SD = 3.18). Approximately 70% scored 26 or above, indicating no cognitive impairment (Nasreddine et al., 2005). In terms of loneliness, 30% were lonely as assessed by the CES-D item, while 46% were identified as lonely based on the NFLM (score ≥ 1). Most participants were White persons, with approximately 9% being Black and close to 6% being Latino. The overall mean sample age was 63.49 years (SD = 9.2) and most participants were female (52%). Overall, 72% of participants were married or living with a partner. Mean perceived economic position was 3.03 (SD = 0.97), with close to half of participants feeling that their economic position was “average” relative to other Americans. Overall, 14% reported missing medical care or medication due to inadequate insurance. The perceived discrimination score was 2.87 (SD = 2.73), indicating that on average, participants felt they were treated with less courtesy or that others acted as if they were better than them less than once a year. Perceived discrimination was higher for Black individuals, with a mean of 3.28 (SD = 2.88) indicating that they felt discriminated close to several times a year. On average, participants reported 1.33 chronic diseases (SD = 1.21) and a mean depressive symptoms score of 7.59, (SD = 3.08). Community participation was 9.21 (SD = 4.22), indicating that on average participants engaged in volunteer work, attended of social meetings, and gathered with friends or relatives several times a year.

In bivariate analyses, CES-D loneliness, NFLM loneliness score, being Black, being Latino, separated, divorced, never married, widowed, skipping healthcare services, having one or more chronic diseases, and having more depressive symptoms were all inversely associated with cognitive functioning. Being female, greater perceived economic position, more community participation, and living alone were all positively associated with higher cognitive scores. Lower educational attainment was also associated with lower MoCA scores.

### Relationship between loneliness and cognitive functioning measures

Table 2 includes two separate adjusted ordinary least squares models estimating the main effects of being lonely based on the CES-D (Model 1) and NFLM loneliness score (Model 2) on MoCA scores. In Model 1, compared to those who reported no loneliness during the past week, CES-D loneliness was inversely associated with global cognitive functioning scores. In Model 2, NFLM loneliness was not associated with MoCA scores. In both models, being Black or Latino, older age, and more depressive symptoms were inversely associated with MoCA scores. Being female and greater community participation were positively associated with higher MoCA scores. Lower educational attainment contributed to worse MoCA scores. Finally, widowhood was inversely associated with MoCA scores in Model 2 only.

### Differences across lonely only groups

The relationship between loneliness measures was significant, *X*^2^(1, *N* = 2,757) = 435.493, *p* < 0.001. Only 40% of lonely individuals were identified as lonely on both measures. In original and nested analyses, there were consistent significant differences in the categories of “CES-D lonely only” and “NFLM lonely only.” Compared to those who were NFLM lonely only, individuals who were CES-D lonely only evidenced significantly lower MoCA and perceived discrimination scores. The CES-D lonely only group also had a greater proportion of Latino participants widows and greater depressive symptoms. We note that in our original analyses (non nested), there were marginally significant differences in chronic disease across groups. In nested analyses, these differences were no longer significant.

### Exploratory analyses: relationship between loneliness and MoCA scores by race and ethnicity

Our exploratory analyses indicated that the association of CES-D loneliness with MoCA score did not vary by race and ethnicity. Figure 1 demonstrates that NFLM loneliness score was not significantly related to cognitive functioning scores for White or Black participants. However, NFLM loneliness was positively associated with better MoCA scores for Latino participants only (βˆ = 0.445, SD = 0.128, *p* < 0.001).

## Discussion

Our study examined the relationship between loneliness and cognitive functioning in a US sample of midlife and older adults community-dwelling Black, Latino, and White adults. After adjusting for multiple salient variables, our results indicated that loneliness identified with the CES-D loneliness item was inversely associated with MoCA scores. However, there was no significant relationship between NFLM loneliness and MoCA scores among White or Black individuals. Counterintuitively, NFLM loneliness was positively associated with better MoCA scores among *Latino participants only.* These findings contribute to the literature examining effects of loneliness on cognitive functioning. Given the subjective nature of loneliness and racial and ethnic diversity in the US context, our findings spark multiple questions for future work.

We hypothesized that both of our measures of loneliness (CES-D and NFLM) would be associated with poorer cognitive functioning. Our findings provided partial support for these hypotheses. Our findings documenting an inverse relationship between CES-D loneliness and global cognitive functioning are consistent with prior research (Boss et al., 2015; Donovan et al., 2017; Poey et al., 2017; Yu et al., 2023). Our final models indicated that NFLM loneliness was not associated with cognitive functioning among Black and White participants. These results are different from cross-sectional examinations by Griffin et al. (2020) and Ishikawa et al. (2022) using the UCLA loneliness scale with predominantly White and Black persons in analyses of the HRS and NSHAP (Griffin et al., 2020; Ishikawa et al., 2022). We consider multiple factors that may explain our findings.

First, our findings suggest that “direct assessment” (e.g., CES-D loneliness) and “indirect assessment” of loneliness (NFLM) capture varying impacts of loneliness on cognitive functioning (Shiovitz-Ezra and Ayalon, 2012). It is possible that the CES-D loneliness item, compared to the NFLM, is a better method for detecting distressing feelings of loneliness and their impact on cognitive functioning among younger (e.g., midlife) and healthier (e.g., no significant cognitive impairment) samples. Although our loneliness measures were significantly correlated, only 40% of lonely individuals were identified as lonely by both measures. We posit that our loneliness measures captured lonely groups with different characteristics that may contribute to health status, levels of stress, and cognitive functioning. Specifically, compared to individuals in the NFLM lonely group, the CES-D lonely group was more likely be widowed and have depressive symptoms? Each of these factors may contribute to overall poorer health (e.g., Kung, 2020) higher stress levels (e.g., Cohen et al., 2016) and may moderate the relationship between loneliness and cognitive functioning.

Second, we consider that the CES-D or NFLM measures of loneliness do not capture the complexity of the experience of loneliness (Kidambi and Lee, 2020). For example, stigma (Lau and Gruen, 1992; Rokach, 2018) associated with the experience of loneliness may lead some individuals to not report their true feelings in response to the CES-D loneliness item. Further, individuals who are assessed as lonely on scales like the NFLM may not believe themselves to be lonely. For example, in a qualitative study of loneliness with older Latino adults (Camacho et al., 2021), some participants cited feeling lonely due to missing loved ones who live outside of the US or deceased loved ones. At the same time, these participants reported being surrounded by close others. Some participants did not feel “left out,” a “lack of companionship,” or “isolated” (Payne et al., 2014) or used these terms to describe their feelings of loneliness. We note that the CES-D lonely group only had higher proportion of Latino participants. Therefore, it is possible that the ability of different assessments to identify lonely individuals may vary across racial and ethnic groups.

Experiences of loneliness likely vary across individuals as a function of clinically relevant dimensions (e.g., frequency of symptoms, chronicity, intensity), but these are not captured by either loneliness assessment employed in the current study. Recently, Yu et al. (2023) noted the significant cumulative effects of loneliness on memory by summing the number of waves in which participants acknowledged feeling lonely on the CES-D item (i.e., during the past week). On the one hand, it is possible that individuals who reported feeling lonely across waves may approximate individuals with chronic loneliness. However, their approach may also include individuals who experienced multiple bouts of situational loneliness across waves. Measures that capture the chronicity of loneliness are necessary to enhance understanding of how they relate to stress and cognitive functioning (Kidambi and Lee, 2020). Further, different types of loneliness (social, emotional, existential; Weiss, 1973) may have differing impact on stress and cognitive functioning on US midlife and older adults. Interestingly, our CES-D lonely only group had a greater proportion of widowed individuals, while those found to be lonely via the NFLM measure reported significantly greater perceived discrimination scores. We posit that perceived causes of loneliness may vary across and within CES-D and NFLM lonely groups, producing varied types of loneliness, chronicity, impact on stress and ultimately cognitive functioning.

We note that multiple factors may explain why our results indicated that NFLM loneliness was not associated with cognitive functioning for Black and White participants.

We consider that the elements of study design and participants’ ages may explain this finding. For example, Griffin et al.’s (2020) cross-sectional analyses yielded a significant relationship between loneliness (UCLA loneliness scale) and cognitive functioning. However, their sample was composed of individuals 65 years and older. Further, our sample included individuals who completed the full MoCA battery and therefore, individuals who were more severely impaired (i.e., unable to answer questions with possible dementia) were not included. As a result, our sample of Black and White participants may have been healthier than prior studies, therefore making it less likely for an effect of NFLM loneliness on cognitive functioning to be detected. Additionally, our study was cross-sectional. Prior studies indicating loneliness (UCLA loneliness scale; De Jong-Giervald Loneliness Scale) is inversely associated with cognitive functioning followed samples over 6 to 10 years on average (Wilson et al., 2007, 2015; Griffin et al., 2020; Sutin et al., 2020). Further, samples from Wilson et al. (2007, 2015) had mean ages in the 80s. Our sample ages ranged from 50 to 97 years old (mean: 63 years). It is possible that the association of loneliness assessed with the NFLM and similar measures is most noticeable in Black and White participants with advanced ages using longitudinal designs.

To date, most US research on loneliness and its impact on health has focused on White adult majority groups. Our inclusion and examination of Black and Latino persons extends the limited literature on loneliness and cognitive health in these important groups (Ojembe et al., 2022; Tibirica et al., 2022). Our exploratory analyses indicated that the effects of CES-D loneliness on cognitive functioning did not vary by race and ethnicity. We acknowledge that our sample of Black and Latino participants lacked the statistical power to capture the moderating effects of race and ethnicity on CES-D loneliness and cognitive functioning (e.g., overall Latino participants with loneliness [direct measure] made up only 2% of the entire sample). However, these same statistical concerns do not apply to our interactions of NFLM scores by race and ethnicity. We found that NFLM loneliness was not associated with cognitive functioning for Black participants. However, NFLM loneliness contributed to better cognitive functioning for Latino individuals in fully adjusted models. Considering these exploratory analyses and findings, we note multiple factors that may explain these findings.

Subjective perceptions, and personal resources such as coping strategies may vary across individuals, particularly across racial and ethnic groups, and partially explain our findings (Pearlin and Schooler, 1978; Lazarus and Folkman, 1984, p. 142; Ferraro and Shippee, 2009). For example, as with other historically oppressed groups, midlife and older Black persons may be better equipped to accept aging challenges as a result of “crisis competence” (Friend, 1991). Years of successful management of one or multiple stigmatized minority statuses earlier in life may influence subjective perceptions of loneliness that enhance their ability to cope with (or suppress) personalized psychological challenges. It will be critical to examine how midlife and older Black individuals perceive and cope with loneliness to better understand the relationship between loneliness, stress, and cognitive functioning.

Interestingly, our exploratory analyses indicated that NFLM loneliness was positively associated with cognitive functioning for Latino participants only. These results contrast with findings from Pluim et al. (2023) and Sutin et al. (2020). Sutin et al. (2020) noted that UCLA loneliness scale scores significantly increased prior to the onset of dementia over a 6-year period for Hispanics from the HRS. However, Pluim et al. (2023) found that loneliness was not associated with subjective cognitive decline for Latino individuals. We underscore that Latino persons are not well represented in studies of loneliness and cognition (Boss et al., 2015; Lara et al., 2019; Tibirica et al., 2022). We considered the possibility that our fully adjusted models may have blurred the true effects of NFLM loneliness on cognitive functioning in Latino participants. However, we note that simplified models (i.e., controlling for race and ethnicity, NFLM loneliness, and interaction) indicated a positive and stronger relationship between NFLM loneliness and cognitive functioning for Latino individuals (βˆ =0.584, *p* < 0.001).

We consider that multiple individual and interacting factors not explored in the NSHAP data may explain our counterintuitive results with Latinos. First, nativity may influence the experience of loneliness and cognitive impairment (Angel et al., 2022; Quiroz et al., 2022). Most Latino participants (63.9%) in our sample were foreign born. We were unable to determine country of origin, years living in the US, and citizenship. We consider that Latinos from different countries or regions of origin, immigration trajectories, and acculturation may have differing exposure to risk and opportunities across the life course (e.g., Puerto Ricans are US citizens) that may contribute to important impacts on loneliness, stress, and cognitive functioning. Nevertheless, migration experiences commonly include feelings of loneliness as they leave behind loved ones and their homeland and join a socially excluded and minoritized group in the US (e.g., Negi et al., 2021). On the one hand, loneliness may be a chronic problem for some participants. On the other hand, some Latino participants in our sample may have found efficient ways of coping or overcoming stress resulting from loneliness. Further, loneliness may even enhance problem solving (e.g., executive functioning), as perceiving limited social resources may push immigrants to identify solutions to everyday challenges with limited or no social support (e.g., lack of family, friends, social services).

Second, we consider that varied availability of contextual resources may influence both the experience and impact of loneliness on cognitive health. For example, living in a Latino enclave can facilitate linguistic interactions and socialization opportunities and healthcare that may protect against loneliness and its effects, particularly for monolingual Spanish speakers (Aranda et al., 2023). Living in ethnic enclaves may also facilitate “active,” “regulative,” and religious or spiritual coping approaches to manage loneliness (Schoenmakers et al., 2012). On the other hand, living in areas where Latinos are a minority may enhance the likelihood of experiencing discrimination and limit opportunities to obtain social support and culturally sensitive health services. These factors may have detrimental effects on feelings of isolation and cognitive health (Viruell-Fuentes and Schulz, 2009).

Third, cultural perspectives are important in the study of loneliness as these may shape the nature and extent of closeness in relationships and social connectedness (Perlman and Peplau, 1981; Van Staden and Coetzee, 2010). Differences between individualist and communal cultures may partially contribute to varying rates, perceptions, experiences, and diverse effects of loneliness on cognitive health among different groups of midlife and older Latinos (Van Staden and Coetzee, 2010). For example, in line with cultural values of familism, as Latinos age in the US, they may wish and expect to maintain close relationships (e.g., live in multigenerational households; Viruell-Fuentes and Schulz, 2009). However, varying levels of acculturation (e.g., acceptance of individualist perspectives) may influence midlife and older Latino adults’ expectations and perceived reality of their relationships with US-born children and extended family, and resulting stress (Garcia Diaz et al., 2019) may impact cognitive functioning.

### Limitations

We recognize several limitations in our study. First, our study was cross-sectional and therefore, we could not establish directionality in the relationship between loneliness and cognitive functioning. Second, our sample included disparate samples across Black, Latino, and White groups. Further work with comparable group sample sizes may further elucidate and confirm possible racial and ethnic differences in the relationship between loneliness and cognitive health. Third, other mental health conditions (e.g., anxiety; Gulpers et al., 2016), life experiences (e.g., grief and loss; Pérez et al., 2018), and treatment (e.g., Puustinen et al., 2011) may influence loneliness, stress processes, and cognitive function. Fourth, our analyses provided an initial glimpse into possible differences in identifying individuals living with loneliness using two common measures of loneliness. Conducting a formal examination of direct and indirect measures of loneliness was beyond the scope of our study. Nevertheless, our findings support the need for formal examinations of psychometric properties of loneliness assessment tools in diverse groups of midlife and older adults. We affirm that midlife and older adult communities include sexual and gender minorities, which was not addressed here. Future research is needed to elucidate how these factors influence the relationship between loneliness, stress, and cognitive health outcomes (Kimberly, 1989; Puustinen et al., 2011; Richardson and King, 2017; Camacho et al., 2018).

## Conclusion: implications for research and practice

Our findings have important implications for research and clinical practice. Our findings suggest that loneliness may lead to lower cognitive functioning, particularly among individuals who are willing to disclose or recognize their feelings of loneliness (i.e., CES-D loneliness). It will be important to focus further attention on understanding how different dimensions of loneliness (e.g., onset, chronicity, frequency) impact cognitive health (global and individual domains). Future research should also test models that can help to explain whether objective and subjective measures of stress mediate or moderate the relationship between loneliness and cognitive functioning. Among diverse groups of midlife and older Black and Latino adults, research is particularly needed to identify coping strategies (e.g., Lazarus and Folkman, 1984; Forrester et al., 2019) and how intra group differences (e.g., immigrant trajectories) influence the relationship of loneliness and cognitive functioning.

Finally, 54% of our sample reported loneliness on at least one measure. Considering that loneliness is associated with multiple negative health outcomes, we underscore a need for loneliness prevention and treatment programs that can be delivered in real-world settings such as primary care, geriatric medicine clinics, and outpatient mental health practices. Most recently, psychological interventions have been shown to be effective in treating loneliness (Hickin et al., 2021). However, given our counterintuitive results with Latinos, future research should explore how cultural and contextual factors may influence loneliness assessment across midlife and older adults Latinos in the US. These examinations may then support further work that examines if and how extant mental health interventions need be adapted to address loneliness across racially and ethnically diverse midlife and older adults (Hickin et al., 2021).

## Data availability statement

The original contributions presented in the study are included in the article or supplementary materials. Further inquiries can be directed to the corresponding author.

## Ethics statement

This study is a secondary analysis of de-identified data. The data were acquired for analysis under a data use agreement from National Archive of Computerized Data on Aging that precluded the investigators from performing analyses that might re-identify participants in the study. Use of the de-identified data for this study was reviewed by the Weill Cornell Medicine Institutional Review Board. The original data were collected by the National Opinion Research Center which obtained informed consent from the participants.

## Author contributions

DC: Conceptualization, Data curation, Formal analysis, Funding acquisition, Investigation, Methodology, Project administration, Software, Writing – original draft, Writing – review & editing. KP: Writing – review & editing. JM: Data curation, Formal analysis, Methodology, Software, Writing – review & editing. MA: Conceptualization, Investigation, Supervision, Writing – review & editing. MR: Conceptualization, Formal analysis, Funding acquisition, Investigation, Methodology, Resources, Supervision, Writing – review & editing. EW: Conceptualization, Formal analysis, Funding acquisition, Investigation, Methodology, Supervision, Writing – review & editing.
