# Corrigendum: Loneliness and global cognitive functioning in racially and ethnically diverse US midlife and older adults

**DOI:** 10.3389/fpsyg.2024.1501339

**Published:** 2024-10-07

**Authors:** David Camacho, Kelly Pacheco, Jerad Moxley, Maria P. Aranda, M. Carrington Reid, Elaine Wethington

**Affiliations:** ^1^Department of Disability and Human Development, University of Illinois Chicago, Chicago, IL, United States; ^2^Weill Cornell Medicine, Cornell University, New York, NY, United States; ^3^USC Suzanne Dworak-Peck School of Social Work, University of Southern California, Los Angeles, CA, United States; ^4^Edward R. Roybal Institute on Aging, University of Southern California, Los Angeles, CA, United States

**Keywords:** Alzheimer's Disease and related dementias (ADRD), cognitive impairment (CI), perceived social isolation, minority aging, African American (AA), Hispanic and Latinx

In the published article, an author name was incorrectly written as Cary Reid. The correct spelling is M. Carrington Reid.

The authors apologize for this error and state that this does not change the scientific conclusions of the article in any way. The original article has been updated.

